# A Novel Role for the Regulatory Nod-Like Receptor NLRP12 in Anti-Dengue Virus Response

**DOI:** 10.3389/fimmu.2021.744880

**Published:** 2021-12-09

**Authors:** Xingyu Li, Zhuo Dong, Yan Liu, Weifeng Song, Jieying Pu, Guanmin Jiang, Yongjian Wu, Lei Liu, Xi Huang

**Affiliations:** ^1^ Center for Infection and Immunity and Guangdong Provincial Key Laboratory of Biomedical Imaging, The Fifth Affiliated Hospital of Sun Yat-sen University, Zhuhai, China; ^2^ Zhongshan School of Medicine, Sun Yat-sen University, Guangzhou, China; ^3^ Department of Clinical Laboratory, The Fifth Affiliated Hospital of Sun Yat-sen University, Zhuhai, China; ^4^ Department of Pharmacy, The Fifth Affiliated Hospital, Sun Yat-sen University, Zhuhai, China; ^5^ National Clinical Research Center for Infectious Diseases, Shenzhen Third People’s Hospital, Southern University of Science and Technology, Shenzhen, China

**Keywords:** DENV, NLRP12, PRDM1, HSP90, type I interferon

## Abstract

Dengue Virus (DENV) infection can cause severe illness such as highly fatality dengue hemorrhagic fever (DHF) and dengue shock syndrome (DSS). Innate immune activation by Nod-like receptors (NLRs) is a critical part of host defense against viral infection. Here, we revealed a key mechanism of NLRP12-mediated regulation in DENV infection. Firstly, NLRP12 expression was inhibited in human macrophage following DENV or other flaviviruses (JEV, YFV, ZIKV) infection. Positive regulatory domain 1 (PRDM1) was induced by DENV or poly(I:C) and suppressed NLRP12 expression, which was dependent on TBK-1/IRF3 and NF-κB signaling pathways. Moreover, NLRP12 inhibited DENV and other flaviviruses (JEV, YFV, ZIKV) replication, which relied on the well-conserved nucleotide binding structures of its NACHT domain. Furthermore, NLRP12 could interact with heat shock protein 90 (HSP90) dependent on its Walker A and Walker B sites. In addition, NLRP12 enhanced the production of type I IFNs (IFN-α/β) and interferon-stimulated genes (ISGs), including IFITM3, TRAIL and Viperin. Inhibition of HSP90 with 17-DMAG impaired the upregulation of type I IFNs and ISGs induced by NLRP12. Taken together, we demonstrated a novel mechanism that NLRP12 exerted anti-viral properties in DENV and other flaviviruses (JEV, YFV, ZIKV) infection, which brings up a potential target for the treatment of DENV infection.

## Introduction

Flaviviruses are a group of enveloped viruses with positive-sense, single-stranded RNA genomes (1). Common flavivirus genus, such as Dengue virus (DENV), Zika virus (ZIKV), Japanese Encephalitis virus (JEV) and Yellow Fever virus (YFV), were widely spread in the global population and caused substantial morbidity each year (2). Of these, DENV remains one of the most widespread flavivirus with an estimated 400 million new infections each year, which contains 100 million clinically apparent cases (3). The DENV presents mainly four different serotypes antigenically, DENV1-DENV4. Infections with any of the DENV serotypes may result in disease, which is primarily characterized by high fevers, severe joint and muscle pain, and rash (4). At worse, if patients suffer a second infection with another serotype, they may result in severe disease such as DHF and DSS, which are accompanied by thrombocytopenia, vascular leakage, and hypotension thus may result in death (5, 6). Although DENV is widespread in various geographical locations, isolation treatment and symptomatic supportive treatment are considered as the major treatment methods, and no specific treatments are available to date (2). Therefore, it is a desperate need for improved understanding of the immunological mechanisms driving DENV infection and developing new therapies.

DENV is transmitted mostly *via* mosquito bites. Mosquitoes maintain productive flavivirus infection and transmit the virus to humans through bites (7). After entering the body, DENV mainly infects myeloid cells in blood and tissues, where some viral components are recognized by pathogen recognition receptors (PRRs), after which the innate immune system is activated (8, 9). TLR-3 and RIG-I are considered to be the primary PRRs involved in DENV recognition (10). TLR-3 recognizes DENV RNA, resulting in phosphorylation of TRIF, which interacts with both TNF receptor associated factor (TRAF) 3 and 6. Through respective association with TANK binding kinase (TBK-1) or transforming growth factor-β-activated kinase 1 (TAK1), TRAF3 and TRAF6 activate nuclear translocation of IRF3, AP-1 and NF-κB thus induce production of IFN-α/β, ISGs and chemokines (11–13). RIG-I recognizes the 5` region of the DENV genome and is considered to be the main sensor for DENV2 and DENV4 in HEK293 cells (14). During DENV infection, TLR-3 and TLR7/8 agonists can maintain TLR-mediated responses and repressed viral replication, which indicated a protective role for PRRs in DENV infection (15). A recent study has also demonstrated that TLR2 along with CD14 and TLR6, can function as a sensor of DENV particles, which induced inflammatory cytokine secretion and impaired vascular integrity (16). These studies illustrated that PRRs play important roles in the host defenses against DENV infection. The nucleotide-binding and oligomerization domain (NOD)-like receptors (NLRs) are a family of cytosolic PRRs, which consists of more than 20 members in humans (17, 18). NLRs are multi-domain proteins composed of an N-terminal effector domain pyrin domain (PYRIN), a central nucleotide-binding and oligomerization domain (NACHT), and a C-terminal leucine-rich repeat domain (LRRs) (19). Some NLRs have been suggested to be involved in the antiviral immunity, and studies have begun to elucidate the involved mechanisms. Several NLRs can form inflammasome complex, which active caspase-1 to cleave pro-IL-1β and pro-IL-18 to their mature forms, such as NLRP1 and NLRP3 (20, 21). Except for the formation of inflammasome, Li et al. demonstrated that the negative regulator NLRC3 directly interacted with viral DNA through its LRR domain, which liberated STING and activated the interferon pathway (22). Another member, NLRC5, has also been reported to be a negative regulator in virus infection, which directly interacted with IKKα/IKKβ and inhibited their phosphorylation thus repressed NF-κB-dependent responses (23). In human hepatocytes, NLRX1 restricted the infection of Hepatitis virus by suppressing IRF3 activation which promoted IRF1 expression and antiviral cytokine production (24). NLRP12 is an important member of the NLRs family, which has been shown to function as both a negative regulator of cytokine production and a component of inflammasome. It has been reported that NLRP12 could associate with tripartite motif 25 (TRIM25) and reduce the ubiquitination of RIG-I, which prevented the interaction of RIG-I and MAVS thus inhibiting the secretion of IFNs and TNF (25). A recent study showed that viral proteases PLpro and 3CLpro of SARS-CoV-2 cause proteolytic cleavage of NLRP12, indicating the important role of NLRP12 in virus-specific immune responses (26).

In this paper, we investigated a novel mechanism of NLRP12 in innate response during DENV infection. NLRP12 suppressed virus replication dependent on its NACHT domain and its interaction with HSP90. In addition, NLRP12 led to increased production of type I IFNs thus limited DENV replication. In summary, this finding sheds light on the mechanism of NLRP12 in inhibiting viral replication, which provided a potential target for therapeutic strategies aimed at controlling flavivirus infection especially DENV infection.

## Materials and Methods

### Ethics Statement

This research was favorably approved by the Sun Yat-sen University ethics committee. All participants provided written informed consent prior to study participation. The samples contained 20 patients with DENV infection (female to male ratio 11: 9; age range 18-70 years) and 20 healthy controls (female to male ratio 11: 9; age range 18-70 years).

### Virus Production and Infection

Dengue-2 virus New Guinea C strain were provided by the Guangzhou Center for Disease Control, and propagated in C6/36 cells. Briefly, C6/36 cells were seeded (1.5 × 10^6^ cells per well) in a six-well plate, after overnight culture at 37°C in a tissue culture incubator, the old medium was removed, and cells were washed twice with phosphate buffered saline (PBS), after which serum-free medium (500μL) and virus stock solution (10μL) were added. Following incubation for 1 hour with shaking every 15 minutes, cultures were supplemented with 1.5 mL standard RPIM1640-medium containing with 10% fetal bovine serum (FBS) and 100 μg/ml penicillin/streptomycin. When more than 90% of the cells showed cytopathic changes (after approximately 2-3 days), the supernatants were carefully collected and viral titers were determined by measuring the 50% tissue culture infective dose (TCID50). For virus infection, cells were plated and cultured for overnight. The amount of virus was calculated according to the formula: MOI = (viral titer × volume of virus)/cell number. Post inoculation, cells were infected in serum-free medium for 1h, and the medium was then replaced with the medium supplemented with 2% FBS and 1% penicillin/streptomycin. The treated cells were cultivated in a cell culture incubator for further experiments.

### PBMCs Isolation and hMDMs Induction

Human PBMCs were isolated from heparin anticoagulated blood by density gradient centrifugation using lymphocyte separation medium (TBD sciences, Tianjin, China). Briefly, the blood was diluted 1:2 with RPMI 1640 medium and layered over the lymphocyte separation medium. The mixture was centrifuged at 1000×g for 20 minutes and the PBMC-containing layer was collected and washed 2 times in cold PBS to remove platelets. For human monocyte-derived macrophages (hMDMs), CD14^+^ monocytes were sorted by negative selection using human naive CD14^+^ monocyte enrichment set (BD Bioscience, CA, USA). The isolated cells were cultured in RPMI 1640 medium containing 10% FBS, 1% penicillin/streptomycin and GM-CSF (10 ng/mL). The culture medium was half-replaced every 3 days. After 7 days of incubation, macrophages were harvested and used in further experiments.

### Real-Time PCR

Total RNA was isolated using TRIzol (Invitrogen, CA, USA) and precipitated with isopropanol. Briefly, cells were lysed in 500μL TRIzol and incubated on ice for 15 min. The samples were then homogenized and prior to being mixed with 200 μL chloroform and incubated at room temperature (RT) for 3 min. The mixture was centrifuged for 15 minutes at 12000 × g at 4°C, and the supernatant was transferred into a new micro-centrifuge tube and precipitated by adding the same volume of isopropanol. After centrifugation, the pellet was washed with cold 70% ethanol, air dried, and resuspended in 30 μL DEPC treated (RNase free) water. Final RNA concentration was determined using Nanodrop 2000 (NanoDrop, DE, USA). For cDNA generation, an iScript cDNA Synthesis Kit (Bio-rad, CA, USA) was used according to its instruction. Real-time PCR assay was performed using SsoFast™ EvaGreen^®^ Supermix (Bio-Rad, CA, USA) on Bio Rad CFX96 Touch real-time PCR system. Simultaneous quantification of β-actin mRNA was used as an internal control. The sequences of q-PCR primers are listed in **Table 1**.

**Table 1:** Sequences of q-PCR primers.

Primer	Sequence
hNLRP12-F	GCAGCTCTCATAGCCAATAAG
hNLRP12-R	CTGACACTTCCTCAACTGAATC
hPRDM1-F	AGGATGCGGATATGACTCTGT
hPRDM1-R	AACCTCTTCACTGTTGGTGGC
DENV-F	TCCTAACAATCCCACCAACAGCA
DENV-R	AGTTCTGCGTCTCCTGTTCAAGA
hIFNα-F	AGAAGGCTCCAGCCATCTCTGT
hIFNα-R	TGCTGGTAGAGTTCGGTGCAGA
hIFNβ-F	CTTGGATTCCTACAAAGAAGCAGC
hIFNβ-R	TCCTCCTTCTGGAACTGCTGCA
hViperin-F	CCAGTGCAACTACAAATGCGGC
hViperin-R	CGGTCTTGAAGAAATGGCTCTCC
hTRAIL-F	TGGCAACTCCGTCAGCTCGTTA
hTRAIL-R	AGCTGCTACTCTCTGAGGACCT
hIFITM3-F	CTGGGCTTCATAGCATTCGCCT
hIFITM3-R	AGATGTTCAGGCACTTGGCGGT
hβ-actin-F	GCTCCTCCTGAGCGCAAG
hβ-actin-R	CATCTGCTGGAAGGTGGACA

### Western-Blot

Cells were lysed using lysis buffer containing 1% (v/v) protease inhibitor cocktail, 1 mM Phenylmethylsulfonyl fluoride, and 1 mM DTT. Total proteins were extracted by centrifugation at 12000 × g at 4°C for 15 min. Input proteins were quantified using Coomassie brilliant blue (Sigma-Aldrich, MO, USA) staining and denatured using 5 × loading buffer and boiled for 10 min. For electrophoresis, equal amount of the proteins was loaded and subjected to SDS-PAGE, after which the proteins were electrically transferred to a PVDF film (Amersham, Ayesbury, UK). The membranes were then blocked in TBST (pH 7.4, 0.5% Tween20) with 5% skim milk powder for 2 hours, and then incubated with the primary antibodies in TBST-5% BSA at 4°C overnight. Then, the membranes were washed with TBST and incubated with secondary antibodies at RT for 1 h, after which the membrane were visualized with an ECL kit (KeyGEN, Nanjing, China). Antibodies were purchased from the indicated companies and used at dilution 1:1000, including primary antibody: rabbit anti-NLRP12 (ab105409, Abcam), rabbit anti-β-actin (13E5, CST), mouse anti-Dengue virus NS1 (SQab1501, Arigo), mouse anti-Dengue virus NS5 (SQab19173, Arigo), rabbit anti-PRDM1 (C14A4, CST), rabbit anti-Flag (D6W5B, CST). Secondary antibody: anti-rabbit IgG mAb (L27A9, CST), anti-mouse IgG mAb (D3V2A, CST).

### Immunofluorescence

THP1 cells were inoculated and treated in confocal dishes. Cells were then treated with PMA (100 nM) for 16 hours, and used as differentiated THP1 (dTHP1) cells. The treated cells were fixed with 4% paraformaldehyde for 30min and permeabilized with 0.1% Triton X-100 in PBS and 0.5% BSA for 30 min. Primary antibody incubation was performed at 4°C overnight with 5% BSA, 0.3% Triton X-100 in PBS. After being thoroughly washed with PBS, corresponding secondary antibodies were added and incubated for 2h at RT. Nuclei were subsequently visualized with DAPI (1:2000) (CST, 4083) and cells were imaged and quantified using the laser confocal microscope (LSM880; Zeiss, Germany). Primary antibodies used were as follows: rabbit anti-NLRP12 mAb (Signalway Antibody, 37750), goat anti-PRDM1 mAb (Thermo fisher, PA5-19216), mouse anti-Dengue virus Envelope (E) protein antibody (Arigo, SQab19170), goat anti-HSP90 antibody (R&D, AF3775). Secondary antibodies used were as follows: Alexa Fluor 594 rabbit anti-goat (Invitrogen, A27016), Alexa Fluor 488 donkey anti-rabbit 488 (Invitrogen, A21206), Alexa Fluor 647 goat anti-mouse (Invitrogen, A32728).

### Plasmid Constructs

Plasmid constructions were performed according to standard procedures using the eukaryotic expression vector PSG5. Briefly, the full-length, deletion fragments and different domains of NLRP12 were amplified and inserted into a multi-cloning site of the PSG5 vector. The mutation of NLRP12 walker A/B motif were achieved through in-fusion PCR cloning. Sequencing verified all plasmid constructs to exclude mutations. All primers used for cloning are described in **Table 2**.

**Table 2:** Sequences of cloning primers.

Primer	Sequence	Enzymes
FLAG-WT-NLRP12-F	CGGGGTACCATGCTACGAACCGCAGGCAG	KpnI
FLAG-WT-NLRP12-R	CCCAAGCTTTCACTTATCGTCGTCATCCTTGTAA TCACCACCACCGCAGCCAATGTCCAAATAAG	HindIII
FLAG-△PYRIN-NLRP12-F	CGGGGTACCATGACCTACAGGGACTATGTCC	KpnI
FLAG-△PYRIN-NLRP12-R	CCCAAGCTTTCACTTATCGTCGTCATCCTTGTAA TCACCACCACCGCAGCCAATGTCCAAATAAGG	HindIII
FLAG-△NACHT-NLRP12-F-1	CGGGGTACCATGCTACGAACCGCAGGCAGG	KpnI
FLAG-△NACHT-NLRP12-F-2	GGTGGCGGTGGAAGCGGCGGTGGCGGAAGCGGC GGTGGCGGCAGCATGGACGAGGGGGAGGGCGGGGC	HindIII
FLAG-△NACHT-NLRP12-R-1	GCTGCCGCCACCGCCGCTTCCGCCACCGCCGC TTCCACCGCCACCTTCCTGGGGATCTTTTCTTGG	KpnI
FLAG-△NACHT-NLRP12-R-2	CCCAAGCTTTCACTTATCGTCGTCATCCTTGTAA TCACCACCACCGCAGCCAATGTCCAAATAAGG	HindIII
FLAG-△LRRs-NLRP12-F	CGGGGTACCATGCTACGAACCGCAGGCAGG	KpnI
FLAG-△LRRs-NLRP12-R	CCCAAGCTTTCACTTATCGTCGTCATCCTTGTAA TCACCACCACCCAGGATATAGTACATAGCTGC	HindIII
FLAG-mutA-NLRP12-F	CAAGGCGCGGCAGGGATAGCGGCAGCGATGCTGGCACAC AAGGTG	/
FLAG-mutA-NLRP12-R	CACCTTGTGTGCCAGCATCGCTGCCGCTATCCCTGCCGCG CCTTG	/
FLAG-mutB-NLRP12-F	CTCCTTTTCATCATCGCAGGCTTCGCGGAGCTCAAGCCTTC TTTC	/
FLAG-mutB-NLRP12-R	GAAAGAAGGCTTGAGCTCCGCGAAGCCTGCGATGATGAAA AGGAG	/

Underlined sequences represent restriction sites.

### NLRP12 Knockdown and Overexpression

Human NLRP12 cloned into PSG5 vector was used for overexpression. For NLRP12 knockdown, NLRP12 specific small interfering RNA (siNLRP12) and negative control siRNA (siNC) were purchased from Santa Cruz Biotechnology, Inc. The experimental method was referred to our previous study (27). Initial experiments were performed in 12-well culture plates at 2×10^5^ cells/well in a 1-ml total volume. 3 pmol/well of siRNAs or 800ng of overexpression plasmids were delivered into cells using lipofectamine (TM) 2000 (Lipo2000) (Invitrogen, CA, USA) and serum-free OptiMEM medium (Gibco, Darmstadt, Germany) as per the manufacturer’s protocol. The transfection medium was replaced by the normal medium after 6 hours, and the cells were continually cultured for 24 hours for further experiments. siRNA sequences are listed in **Table 3**.

**Table 3:** siRNA sequences.

Gene	Sequence
siNLRP12-1	GAGAGGUACUACAGCUUCA
siNLRP12-2	GCAGGAAAUUCCGGCUCAU
siPRDM1-1	GCAGCATGAATGGCATCAA
siPRDM1-2	GGACCTCGATGACTTTAGA

### Rescue Experiment and Double Knockdown Experiment

The method of this experiment was referred to the previous research (28). PRDM1 rescue experiment was conducted in NLRP12-FLAG-expressing dTHP1 cells which were transfected with siRNAs. In brief, dTHP1 cells were transiently transfected siRNAs with by Lipo2000 (Invitrogen, CA, USA) for 24h, then PRDM1-Flag-expressing vectors were transfected into the cells using Lipo2000 (Invitrogen, CA, USA) for 24h. After 72h post-infection, the cells were harvested for western blot analysis. NLRP12 rescue experiment were similar to those described in PRDM1 rescue. Double knockdown experiments were also conducted in dTHP1 cells. The method of this experiment was referred to the previous research and 10 nM of each siRNA were transfected simultaneously (28).

### Statistical Analysis

Data processing and analyses were performed in GraphPad Prism 5.0 Software (Graphpad, CA, USA). The statistical significance was determined with analysis of one-way analysis of variance (ANOVA). Data are shown as mean ± SD unless otherwise stated. A p-value of < 0.05 was regarded as statistically significant.

## Results

### NLRP12 Expression Was Significantly Reduced After DENV Infection

**Figure 1:**
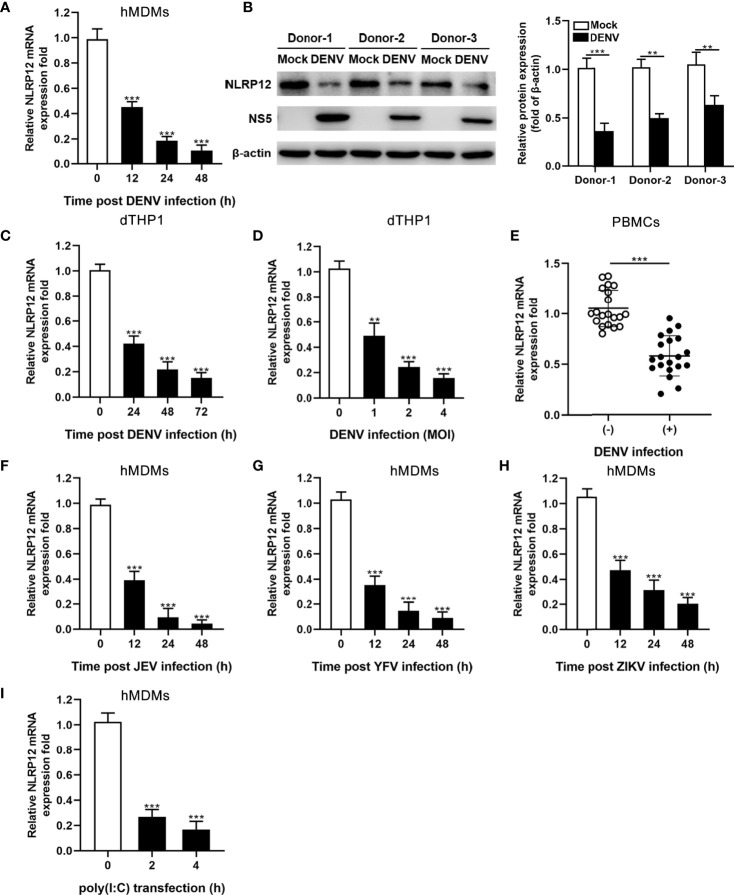
DENV infection significantly down-regulated the expression of NLRP12. **(A)** Monocytes were isolated and induced into hMDMs using GM-CSF. Cells were infected with DENV (MOI=4) for indicated time points. The mRNA expressions of NLRP12 were detected by qPCR. **(B)** hMDMs from 3 different donors were infected with DENV for 48h (MOI=4), NLRP12 protein expression was determined *via* western blot analysis and the protein bands were quantitated by Image J gel image analysis software and the relative grayscale value was normalized to that of β-actin. **(C, D)** THP-1 cells were differentiated using PMA and infected with DENV for indicated time points (MOI=4) and indicated MOI (72h), the level of NLRP12 mRNA were evaluated by qPCR. **(E)** Total RNA of the PBMCs from patients with DENV infection and healthy donors was isolated, and qPCR was used to measure NLRP12 gene expression. **(F–I)** hMDMs were stimulated with JEV, YFV, ZIKV or poly (I:C) respectively for the indicated time (MOI=4), and qPCR was used to measure gene expression level of NLRP12. Each experiment was repeated a minimum of three times and the statistical significance was determined with analysis of one-way analysis of variance (ANOVA). ^**^P < 0.01, ^***^P < 0.001.

To investigate the impact of flaviviruses infection on expression of NLRs, hMDMs were infected with DENV, and NLRs mRNA levels were detected by qPCR. Data showed that DENV infection dramatically reduced the expression of NLRP12 (**Figure 1A**), while no differences were observed in NLRP2, NLRP4, NLRP7 and NLRP10 expression (data not show). In addition, NLRP12 protein level was also decreased in DENV-infected hMDMs (**Figure 1B**). Going further, dTHP1, which expressed high levels of NLRP12, also showed decreased expression of NLRP12 after DENV infection in time- and MOI-dependent manners (**Figures 1C, D**). We also assessed relative abundance of NLRP12 in PBMC samples from patients with DENV infection and healthy donors using qPCR, which collectively suggested low NLRP12 expression in PBMCs of patients with DENV infection (**Figure 1E**). As most flavivirus infection exhibited similar immunological changes, we detected the expression of NLRP12 in hMDMs with other flavivirus (JEF, YFV and ZIKV) infection as well as viral mimic polyinosinic-polycytidilic acid [poly (I:C)] treatment, which verified that reduced expression of NLRP12 is a generalized phenomenon in flavivirus infection (**Figures 1F–I**). Taken together, the results demonstrated that NLRP12 was down-regulated in human macrophage infected with DENV and other flavivirus, such as JEF, YFV and ZIKV.

### DENV Inhibited NLRP12 Expression by Up-Regulating PRDM-1 in a Manner Dependent on the TBK1/IRF3 and NF-κB Signaling Pathways

**Figure 2:**
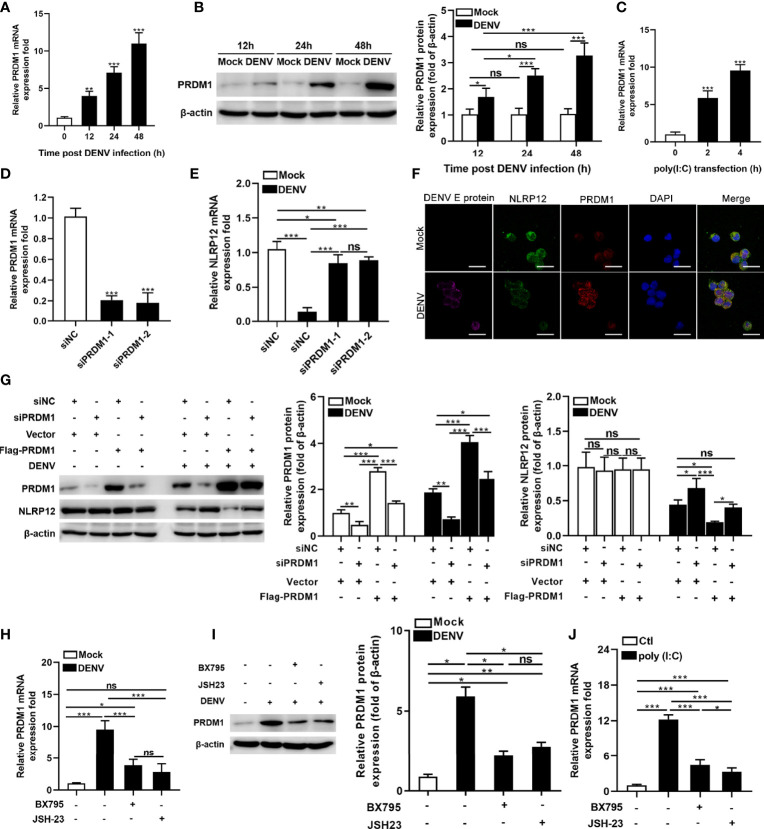
DENV suppressed NLRP12 expression by up-regulating PRDM-1 expression in a manner dependent on the TBK1/IRF3 and NF-κB signaling pathways. **(A, B)** hMDMs were infected with DENV (MOI=4) for indicated time periods and PRDM1 expression was determined by qPCR and western blot. The gray value of PRDM1 protein bands was analyzed by Image J gel image analysis software, and the relative grayscale value was normalized to that of β-actin. **(C)** hMDMs were treated with *Poly*(*I:C*) and harvested for PRDM1 expression analysis. **(D)** The siRNA knockdown of PRDM1 was performed in hMDMs and the mRNA level of PRDM1 was measured. **(E)** PRDM1-silenced hMDMs were infected with DENV for 48h (MOI=4) and NLRP12 expression was determined *via* qPCR. **(F)** dTHP1 cells were infected with DENV with an MOI of 4 for 72h. Immunofluorescence staining was performed for DENV E protein, NLRP12, PRDM1 and DAPI (Bar: 10μm). **(G)** dTHP1 cells were transiently transfected with the siRNAs against the PRDM1, and then PRDM1-Flag-expressing vectors were transfected into the cells. After DENV infection at a MOI of 4 for 72h, the expression levels of NLRP12 and PRDM1 were detected by western blot analysis. The gray value of PRDM1 protein and NLRP12 bands was analyzed by Image J gel image analysis software and the relative grayscale value was normalized to that of β-actin. **(H, I)** hMDMs cells were pre-treated with the inhibitors for TBK1 (BX795) or NF-κB (JSH-23) and subjected to DENV infection at a MOI of 4 for 48h, then the PRDM1 transcript and protein levels were determined. The gray value of PRDM1 protein bands was analyzed by Image J gel image analysis software and the relative grayscale value was normalized to that of β-actin. **(J)** hMDMs cells were pre-treated with the inhibitors for TBK1 (BX795) or NF-κB (JSH-23) and subjected to poly(I:C) treatment, then the PRDM1 transcript level was determined. Each experiment was repeated a minimum of three times and the statistical significance was determined with analysis of one-way analysis of variance (ANOVA). ns, nonsignificant, ^*^P < 0.05, ^**^P < 0.01, ^***^P < 0.001.

Gene expression of NLRP12 is suppressed by PRDM1, which interacted with the promoter of NLRP12 and resulted in a correlative loss of histone acetylation (27, 28). To assess whether DENV regulated NLRP12 expression through controlling PRDM1, we firstly examined the levels of PRDM1 in DENV-infected human macrophage. DENV infection induced the mRNA expression of PRDM1 in a time-dependent manner (**Figure 2A**). Consistent with mRNA expression, PRDM1 protein expression was up-regulated in macrophage in a time-dependent manner (**Figure 2B**). Similar to DENV infection, poly(I:C) also promoted the expression of PRDM1 (**Figure 2C**). To investigate the role of PRDM1 in regulating NLRP12 expression, we utilized siRNA to silence PRDM1 in hMDMs and revealed decreased PRDM1 expression by qPCR (**Figure 2D**). Next, we detected the RNA level of NLRP12 in PRDM1-silenced hMDMs after DENV infection. Consistent with expectation, silencing PRDM1 restored DENV-induced suppression of NLR12 expression (**Figure 2E**). Consistently, the result of confocal microscopy also showed that the expression of PRDM1 increased, whereas the expression of NLRP12 decreased in the context of DENV infection, but it seems that DENV infection did not alter the localization of NLRP12 and PRDM1 (**Figure 2F**). We also carried out the rescue experiment in PRDM1-Flag-expressing dTHP1 cells which were transfected with PRDM1 siRNA in advance. Expression of PRDM1 was markedly decreased following siRNA silencing and was significantly increased after transfection with expression plasmid (**Figure 2G**). When the cells were infected with DENV, the expression of NLRP12 was up-regulated by knockdown of PRDM1 expression and was down-regulated by PRDM1 rescue (**Figure 2G**). However, it seemed that PRDM1 did not affect NLRP12 expression without DENV infection, since no significant change in NLRP12 expression was observed in PRDM1 overexpression or silencing group at resting state (**Figure 2G**). These experimental results suggested that NLRP12 expression was regulated by PRDM1 in macrophage during DENV infection. In addition, previous studies indicated that PRDM1 expression could be regulated by several signaling pathways such as NF-κB signaling pathway and TBK1/IRF3 signaling pathway (29, 30). We next sought to determine if the NF-κB and TBK1/IRF3 signaling pathways were required to regulate the expression of PRDM1 during DENV infection. We found that PRDM1 expression was reduced both in RNA level and protein level after NF-κB inhibitor JSH-23 and TBK1/IRF3 inhibitor BX795 treatment (**Figures 2H, I**). Similar trends were observed in poly(I:C) stimulation (**Figure 2J**). Taken together, we confirmed that the expression of NLRP12 was down-regulated by PRDM1 which was dependent on NF-κB and TBK1/IRF3 activation in DENV infection.

### NLRP12 Suppressed DENV Viral Load in Human Macrophage *In Vitro*

**Figure 3:**
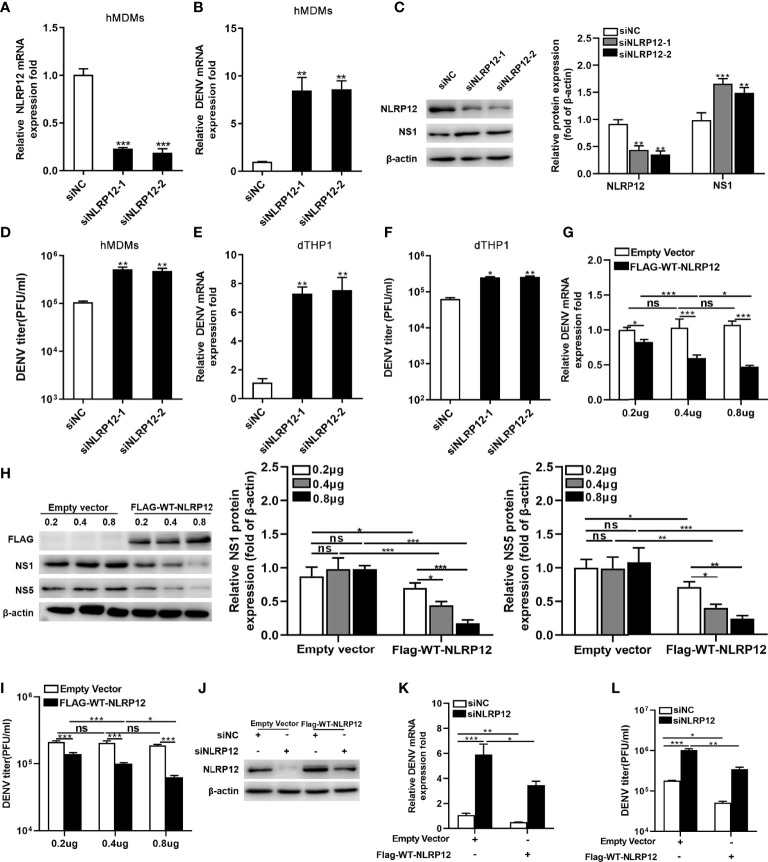
NLRP12 inhibited DENV replication *in vitro*. **(A)** hMDMs were transfected with control siRNA or NLRP12 siRNA and the RNA silencing efficiency of NLRP12 was shown. **(B, C, D)** NLRP12- silenced hMDMs were infected with DENV at a MOI of 4 for 48h, after which the DENV genome RNA levels were detected by qPCR and protein levels of NS1 were assessed respectively by western blot. The gray value of NS1 protein bands was analyzed by Image J gel image analysis software and the relative grayscale value was normalized to that of β-actin. The viral titer in supernatant was quantified by plaque assay. **(E, F)** dTHP-1 cells were transfected with control siRNA or NLRP12 siRNA and infected with DENV at a MOI of 4 for 72h, the DENV genome RNA and the viral titer of supernatant were measured. **(G, H, I)** dTHP1 cells were transfected with overexpression plasmids of NLRP12 and infected with DENV at a MOI of 4 for 72h, after which the levels of DENV genome RNA and NS1 and NS5 proteins were detected. The gray value of NS1 and NS5 bands was analyzed by Image J gel image analysis software and the relative grayscale value was normalized to that of β-actin. The viral titer of supernatant was detected by plaque assay. **(J)** dTHP1 cells were transfected with Flag-tagged NLRP12 plasmid or empty expression vector after which NLRP12 siRNAs were transfected. A portion of the cells were harvested for western blot. **(K, L)** The rest cells were infected with DENV at a MOI of 4 for 72h, then DENV RNA levels and the virus titers in supernatants were assessed. Each experiment was repeated a minimum of three times and the statistical significance was determined with analysis of one-way analysis of variance (ANOVA). ns, nonsignificant, ^*^P < 0.05, ^**^P < 0.01, ^***^P < 0.001.

Previous studies showed that NLRP12 deficiency reduced bacterial burdens in salmonella infection but had no influence in influenza virus load (31, 32). To investigate the impact of NLRP12 on DENV replication, two siRNAs targeting NLRP12 were designed and transfected into hMDMs, the knockdown effect of its mRNA level was about 80% in NLRP12 siRNA transfected hMDMs compared with those transfected with negative siRNA controls (**Figure 3A**). Silencing of NLRP12 increased DENV RNA levels and viral protein NS1 expression in hMDMs (**Figures 3B, C**) and facilitated virus titers in supernatant (**Figure 3D**). Similar results were obtained in dTHP1 cells, in which the RNA levels and titers of DENV in the supernatant were reduced after NLRP12 silencing (**Figures 3E, F**). To confirm the role of NLRP12 in DENV replication, dTHP1 cells were transfected with NLRP12 overexpression plasmid. Reduced DENV viral load was observed concentration-dependently by measurement of viral genomic copy numbers and the viral protein expression in cells as well as the viral titer of supernatants (**Figures 3G–I**). Furthermore, to exclude off-target effects, we transfected siRNAs target NLRP12 in dTHP1 cells and rescued its expression *via* plasmid (**Figures 3J**). The viral RNA level and virus titers were significantly increased in dTHP1 cells with NLRP12 knockdown (**Figures 3K, L**). And when the expression of NLRP12 was rescued, viral RNA levels and virus titers were down-regulated in NLRP12 knockdown dTHP1 cells (**Figures 3K, L**). These observations illustrated regulatory effects by NLRP12 in DENV infection. For another, in the previous section, we elaborated that PRDM1 could inhibit the expression of NLRP12 (**Figure 2**), which allowed for speculation that PRDM1 might promote DENV infection. To validate this conjecture, we explored the role of PRDM1 in DENV replication *via* siRNAs targeting PRDM1 and NLRP12. We found that knockdown of PRDM1 resulted in a decrease in viral RNA levels. No difference was observed in double knockdown group of as compared to the control group (**Figures S1A, B**). These results suggested that NLRP12 suppressed DENV viral load in macrophage.

### NLRP12 Promoted Anti-DENV Responses Dependent on Its NACHT Domain

**Figure 4:**
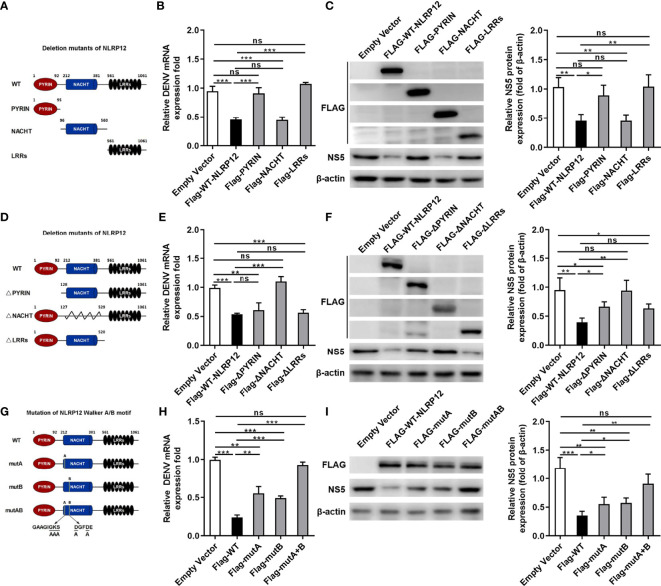
NACHT domain was critical for the functions of NLRP12 in suppressing viral replication. **(A)** Expression plasmids for Flag-tagged NLRP12 truncation mutants were constructed according to the structure of PYRIN domain, NACHT domain and LRRs domain. **(B, C)** The plasmids carrying the Flag-tagged truncations were transformed into dTHP-1 cells, after which the cells were infected with DENV at a MOI of 4 for 72h, qPCR and western blot were performed to determine the levels of genome RNA and the nonstructural proteins NS5.The protein bands were analyzed by Image J gel image analysis software and normalized to β-actin. **(D)** Expression plasmids of NLRP12 lacking the PYRIN domain, the NACHT domain or the LRRs domain were constructed accurately. **(E, F)** Plasmids with deletions were transfected into dTHP-1 cells, after which the cells were infected with or without DENV at a MOI of 4 for 72h and assessed for the levels of genome RNA and the nonstructural protein NS5. The protein bands were analyzed by Image J gel image analysis software and normalized to β-actin. **(G, H, I)** FLAG-tagged NLRP12 with Walker A/B mutations (mutA, mutB and mutA+B) were generated, and each of them was overexpressed into dTHP-1 cells. Following by DENV infection for 72h at a MOI of 4. Cell extracts were collected and the intracellular viral RNA levels, as well as the protein levels of NS5 were determined. NS5 protein bands were analyzed by Image J gel image analysis software and normalized to β-actin. Each experiment was repeated a minimum of three times and the statistical significance was determined with analysis of one-way analysis of variance (ANOVA). ns, non significant, ^*^P < 0.05, ^**^P < 0.01, ^***^P < 0.001.

To explore the role of different domains within NLRP12-mediated anti-DENV response, we constructed the three truncation mutants of NLRP12 based on its domain structure (**Figure 4A**). We transiently transfected these plasmids into dTHP1 cells and infected these cells with DENV, after which the levels of viral RNA and viral protein NS5 was detected. Results showed that only the overexpression of NACHT domain served similar functions with the full-length WT NLRP12, which resulted in low levels of DENV RNA and DENV protein NS5 (**Figures 4B, C**). However, the PYRIN or LLRs domain were not required for the anti-viral function of NLRP12 (**Figures 4B, C**). To validate these results, we also constructed deletion mutants of NLRP12 (**Figure 4D**). When the NACHT domain was removed, the effects of NLRP12 on the replication of DENV was lost but the deletion of PYRIN or LLRs domain almost have no effect on NLRP12-supressed DENV replication (**Figures 4E, F**). These results suggested that it was the NACHT domain but not the PYRIN or LRRs domain that contributed to NLRP12-induced DENV control. The NACHT domain of NLRP12 is reported to be responsible for ATP-dependent oligomerization which contains multiple well-conserved nucleotide binding sites (33). The ATP/GTP-specific phosphate binding loop Walker A motif and the Mg^2+^ coordination site Walker B have been reported to be important for its ATPase activity (33–35). To further study the functions of these well-conserved nucleotide binding structures in DENV infection, we introduced mutations within the Walker A (GAAGIGKS > GAAGIAAA) and Walker B (DGFDE > AGFDE) motifs and transfected them into dTHP1 cells (36) (**Figure 4G**). Mutant of Walker A (mutA) or Walker B (mutB) significantly reduced the inhibitory effect of NLRP12 in DENV replication including mRNA and protein (NS5) expression (**Figures 4H, I**). These results suggested that both well-conserved nucleotide binding structures, Walker A motif and Walker B motif, functioned importantly in controlling DENV and they performed a synergistic effect. Altogether, we demonstrated that the NACHT domain was the most critical domain for NLRP12 to regulate DENV infection, and amino acid substitution mutations further revealed that Walker A and Walker B of NLRP12 played major roles in DENV controlling.

### NLRP12 Interacted With HSP90 to Mediate Regulation of Anti-DENV Responses

**Figure 5:**
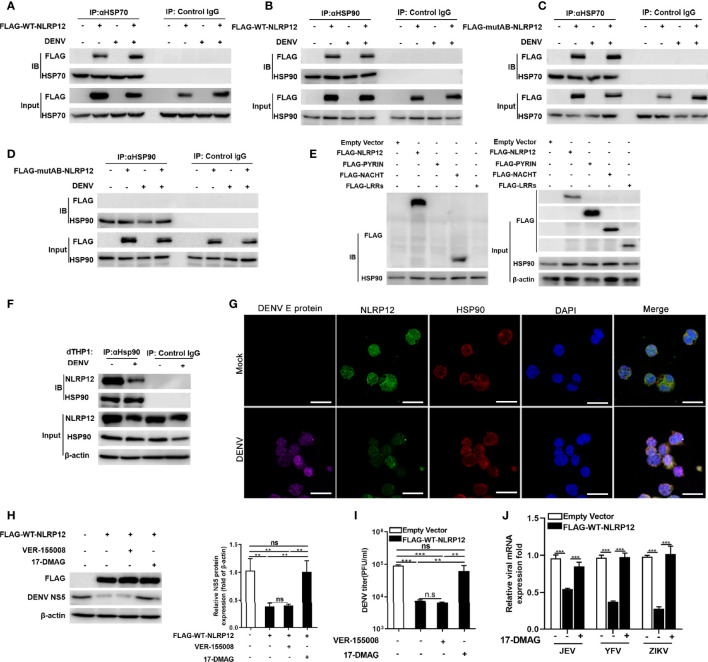
HSP90 interacted with NLRP12 and regulated its antiviral function. **(A, B)** dTHP1 cells were transfected with FLAG-tagged NLRP12 plasmid and infected with or without DENV at a MOI of 4 for 72h, the interaction between exogenous NLRP12 and endogenous HSP70 or HSP90 was detected by Co-IP. **(C, D)** FLAG-tagged NLRP12 Walker A/B mutants (mutA, mutB and mutA+B) were transfected into dTHP-1 cells, and the cells were infected with or without DENV at a MOI of 4 for 72h, the interaction between mutated NLRP12 and HSP70 or HSP90 was detected by Co-IP. **(E)** The plasmids carrying the Flag-tagged full length and three truncations were transformed into dTHP1 cells, after which the cells were collected to detect the interaction between HSP90 and exogenous NLRP12 and its truncations by Co-IP. **(F)** dTHP1 cells were infected with or without DENV (MOI: 4) for 72h. Cells were lysed by RIPA lysis buffer and immunoprecipitated complexes were collected using protein A/G plus agarose beads and analysis by western blotting. **(G)** dTHP1 cells were infected with or without DENV (MOI: 4) for 72h. Immunofluorescence staining was performed for DENV E protein, NLRP12, PRDM1 and DAPI and cells were imaged using confocal microscopy (Bar: 10μM). **(H, I)** NLRP12-transfected dTHP-1 cells were treated with HSP70 inhibitor VER-155008 or HSP90 inhibitor 17-DMAG, the treated cells were infected with DENV at a MOI of 4 for 72h, and the protein level of NS5 was detected by western blot and NS5 protein bands were analyzed by Image J gel image analysis software and normalized to β-actin. The viral titer of supernatant samples was measured by plaque assays. **(J)** NLRP12 overexpressing dTHP-1 cells were treated with HSP90 inhibitor 17-DMAG, cells were then infected with JEV, YFV and ZIKV separately, virus genes were finally detected using quantitative qPCR. Each experiment was repeated a minimum of three times and the statistical significance was determined with analysis of one-way analysis of variance (ANOVA). ns, non significant, **p < 0.01, ***p < 0.001.

HSP70 and HSP90 are heat shock proteins (HSPs) which facilitate the correct folding of other proteins under physiological and stress conditions (37, 38). An increasing number of studies have demonstrated that many NLRs including NOD1, NOD2, NLRP3 and NLRC4 could interacted with HSP70 and HSP90 to achieve proper folding conformation (39–41). To explore the regulation of NLRP12 by HSP70 and HSP90, we detected the interaction between exogenous NLRP12 and endogenous HSP70/90 in dTHP1 cells. Co-IP results showed that both HSP70 and HSP90 could co-precipitate with exogenous NLRP12 before and after DENV infection (**Figures 5A, B**). However, when the Walker A and Walker B motifs were mutated, HSP90 lost its interaction with NLRP12 while HSP70 did not (**Figures 5C, D**). This suggested that NACHT domain was the crucial domain for the interaction between HSP90 and NLRP12, which was further confirmed by Co-IP experiments between HSP90 and the three truncations of NLRP12, in which HSP90 was interacted with NACHT domain but not with the PYRIN or LRRs domain (**Figure 5E**). Endogenous Co-IP and immunofluorescence analysis also suggested physically interacted and colocalization between endogenous HSP90 and NLRP12 (**Figures 5F, G**). Furthermore, treatment of NLRP12-overexpressed dTHP1 cells with the HSP90 inhibitor 17-QMAG resulted in a significant increase in DENV RNA and protein levels (**Figures 5H, I**). However, we did not observe any change in HSP70 inhibitor VER-155008 treated cells (**Figures 5H, I**). HSP90 inhibitor 17-QMAG also restored the NLRP12-induced controlling in JEF, YFV and ZIKV replication (**Figure 5J**). To sum up, these results indicated that NLRP12 regulated the anti-DENV response *via* interaction with HSP90.

### NLRP12 Promoted Type I IFNs Production in a HSP90-Dependent Manner

**Figure 6:**
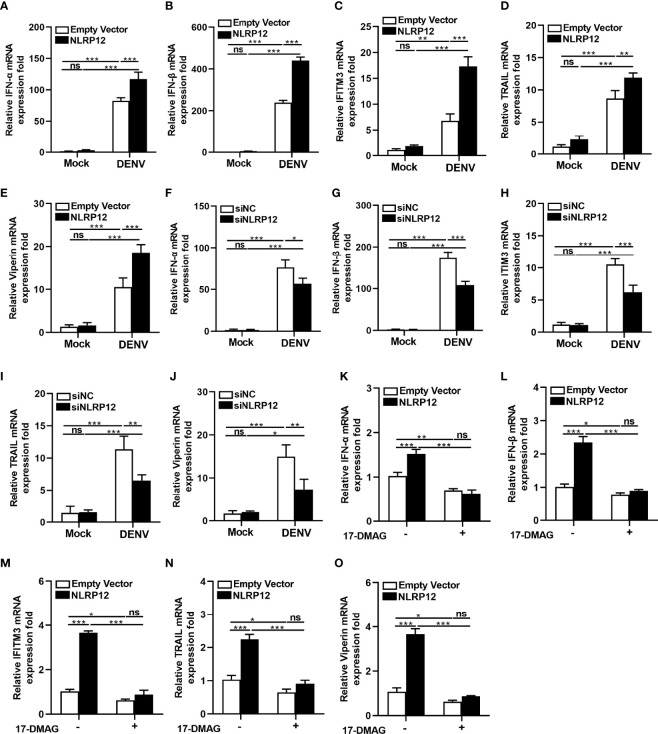
HSP90 and NLRP12 facilitated type I interferon (IFNα/β) production. dTHP1 cells were transfected with FLAG-tagged NLRP12 plasmid or empty vector plasmid. At 24 h post-transfection, cells were infected with or without DENV (MOI: 4) for 72h and the total RNA was isolated and subsequently reverse transcribed. **(A, B)** The mRNA expression levels of IFNs (IFN-α and IFN-β) were measured by qPCR. **(C–E)** The mRNA expression levels of DENV-related ISGs (IFITM3, Viperin or TRAIL) were also measured by qPCR. dTHP1 cells were transfected with control siRNA or NLRP12 siRNA. At 24 h post-transfection, cells were infected with or without DENV (MOI: 4) for 72h and the total RNA was isolated and subsequently reverse transcribed. **(F, G)** The mRNA expression levels of IFNs (IFN-α and IFN-β) and **(H–J)** ISGs (IFITM3, Viperin or TRAIL) were measured by qPCR. **(K–O)** dTHP1 cells were transfected with FLAG-tagged NLRP12 plasmid or empty vector plasmid. At 24 h post-transfection, cells were treated with 1μM 17-DMAG for 24 h and infected with or without DENV. Then the total RNA was extracted and reversely transcribed. The mRNA expression levels of NLRP12, IFNs (IFN-α and IFN-β) and ISGs (IFITM3, TRAIL and Viperin) were measured by quantitative real-time PCR. Each experiment was repeated a minimum of three times and the statistical significance was determined with analysis of one-way analysis of variance (ANOVA). ns, non significant, *p < 0.05, **p < 0.01, ***p < 0.001.

Type I IFNs, including IFN-α and IFN-β, induced expression of interferon stimulated genes (ISGs), which contributed to the antiviral cellular response and prevented viral replication in anti-viral host defenses (29–31). It has been reported that ISGs, including Viperin, TRAIL, IFITM1, IFITM2, IFITM3, ISG20, and TRIM56 could exert efficient antiviral effects in DENV infection (32). Herein, we investigated the regulation of type I IFNs and ISGs by NLRP12. When NLRP12 was overexpressed, the RNA levels of IFN-α and IFN-β showed a significant upregulation in dTHP1 cells (**Figures 6A, B**). This was accompanied by a significant promotion in DENV associated ISGs gene expression, including IFITM3, TRAIL and Viperin (**Figures 6C–E**). Correspondingly, the levels of the IFN-α and IFN-β (**Figures 6F, G**) as well as ISGs including IFITM3, TRAIL and Viperin (**Figures 6H–J**), were markedly reduced in dTHP1 cells with NLRP12 knockdown. Then we focused on the regulation of HSP90 for NLRP12-mediated modulation of type I IFNs and ISGs. Treatment with HSP90 inhibitor 17-DMAG reduced the IFN-α and IFN-β expression induced by NLRP12 overexpression (**Figures 6K, L**), similar to that of IFITM3, TRAIL and Viperin (**Figures 6M–O**). These results might suggest that NLRP12 suppressed DENV replication by promoting type I IFNs production, which was dependent on HSP90.

## Discussion

The involvement of NLRP12 has been demonstrated in a variety of infectious diseases. It was previously shown that *in vitro* knockdown of NLRP12 up-regulated IL-6 and TNF-α expression in bacterial infections, such as salmonella and *Klebsiella pneumonia* infection (33, 34). During *Y. pseudotuberculosis* infection, NLRP12 was an important member for the formation and assembly of inflammasome complex, which promoted IL-1β and IL-18 production (35). In parasites infection, NLRP12 can function in inflammasome formation and was crucial for caspase-1 activation and IL-1β production during rodent malaria infection (36). Several studies also investigated the role of NLRP12 in modulating viral infection. In influenza A virus (IAV) infected mice, NLRP12 exacerbated the pathogenesis of infection by facilitating massive neutrophil chemotaxis *via* promoting CXCL1 expression (37). However, NLRP12 disrupted the interaction between RIG-I and MAVS and enhanced the ubiquitination of RIG-I thus suppressed the production of IFN-β and TNF to facilitated viral replication (25). However, we found that NLRP12 effectively restricted viral replication and promoted type I IFNs and ISGs expression, which relied on the interaction with HSP90 in DENV infection. IFN system is a major branch of innate immunity against DENV and other flavivirus, which plays an important role in controlling virus replication. In addition, the mammalian HSP system, including HSP90, are chaperone proteins that might be directly involved in virus replication. It has been previously reported that HSP90 promoted the formation of the DENV E protein but limited DENV replication (38). Our study suggesteed a new underlying mechanism that HSP90 promoted NLRP12-mediated production of type I IFNs, thus exerted its antiviral effect. These results also illustrated that NLRP12 had a rich diversity and differential immune regulation in infectious disease, which might be pathogen involved.

DENV infection reduced the expression of NLRP12 by promoting PRDM1 transcription, which depended on the activation of NF-κB and TBK1/IRF3 signaling pathways. It is therefore theoretically possible that the DENV component which activated NF-κB and TBK1/IRF3 signaling pathways has the potential to trigger the up-regulation of PRDM1. One possibility is that DENV protease NS2B3 triggers the up-regulation of PRDM1. Since NS2B3 could interact with and cleave IκBα and IκBαβ thus induce NF-κB activation and induce TNF-α production (39). Several studies also pointed out that NS2B3 can regulate IRF3 activation through a protease-dependent mechanism (40, 41). The nonstructural proteins NS5 also have the potential to regulate PRDM1 expression, because it has classically been recognized that NS5 is a positive regulator of the NF-κB pathway (42–44). In addition, some studies also point out that TLR7 and TLR9 ligands could stimulate PRDM1 expression through a Ras-related C3 botulinum toxin substrate (Rac)-mediated pathway in plasmacytoid dendritic cells (pDCs) (45). Since TLR7 is a well-established signaling receptor that recognizes viral RNA, which suggests that DENV genomic RNA might trigger the up-regulation of PRDM1 (46, 47). At the same time, we translated ply(I:C), an analogue of viral RNA, to THP1 cells and observed higher expression of PRDM1, which further indicated the role of viral RNA in PRDM1 expression.

The involvement of PRDM1 in NLRP12 expression have been investigated in skin sensitization and colitis, in which the expression of NLRP12 was also negatively associated with PRDM1 expression (48, 49). This might be because of the interaction between PRDM1 and its binding site in the promotor of NLRP12, which decreased NLRP12 promotor’s activity and expression (50). Consistent with these reports in NLRP12, PRDM1 has also been reported to negatively regulate NF-κB-NLRP3 signaling pathway in *Leishmania donovani* infection. Knockdown of PRDM1 in macrophages reversed NF-κB expression, IFN-γ and TNF-α secretion and cell pyroptosis (51). In addition to the regulatory effects of NLRs, PRDM1 is also known to be involved in T-cell differentiation and function (52). T cells received antigenic stimulation and promoted IL-2 secretion and IL-2R production. After the engagement of IL-2 and IL-2R, PRDM1 expression was increased, which in turn repressed IL-2 expression and T-cell differentiation (53). During chronic viral infection, PRDM1 was highly expressed in CD8^+^ T cells and was positively correlated to the expression levels of inhibitory receptors, which suggested that PRDM1 was a key transcription factor for regulating T cell exhaustion (54). These investigations suggested the complex regulations of PRDM1 in immune response, and our study has provided evidence for PRDM1 to regulate immune response in acute viral infection.

In this study, we revealed for the first time that NLRP12 could interact with HSP70 and HSP90 and regulate IFN-α/β expression and anti-DENV activity, which might depend on the interaction between HSP90 and the Walker A and Walker B motifs. HSP90 is a chaperone protein that can stabilize client proteins and regulate protein kinase activity (55, 56). The client protein of HSP90 can be host proteins. Previous studies found that HSP90 can interact with B-cell Lymphoma 6 (Bcl-6) and maintained the stability of its mRNA, which was required to the survival of B-cell lymphomas cells (57). During breast tumor progression, HSP90 was activated and protected migration inhibitory factor (MIF) from degradation, inhibition of HSP90 destabilized MIF and repressed the tumor growth (58). In the models of the MPN polycythemia vera (PV) and essential thrombocytosis (ET), HSP90 interacted with JAK2 and HSP90 inhibitor (PU-H71) could disrupted the stability of JAK2 protein and reduced lineage-specific myeloproliferation thus inhibited the cell growth in MPN patient samples (59). In addition, HSP90 can form complexes with CDC37 and enhanced types I and II interferons in VSV infection (60). Multiple studies have also indicated that HSP90 could promote the replication of a variety of virus by interacting with viral proteins and maintaining the protein levels. For instance, HSP90 regulated the maturation of the large (L) protein of mumps virus and promoted its replication (61). During chikungunya virus infection, HSP90 stabilized the non-structural protein NSP2 level and enhanced viral replication (62). However, in DENV infection, inhibition of HSP90 led to less formation of the DENV E protein but an increased virus titer, although co-immunoprecipitation assays showed that HSP90 can interacted directly with multiple DENV proteins including E, NS2B, NS3, NS4B and NS5 (38). Here, we found that HSP90 inhibitor (17-DMAG) inhibited NLRP12-induced IFN-α/β expression. Therefore, we conjectured that NLRP12 might be a client protein of HSP90, and the stability of NLRP12 was probably maintained by HSP90. The inhibition of HSP90 might decrease NLRP12 stability thus counteracted the NLRP12-induced anti-DENV effects, which may partly explain that HSP90 enhanced expression of DENV E protein but decreased virus titer.

In conclusion, our data showed that DENV infection reduced NLRP12 expression by inducing PRDM1 expression. NLRP12 promoted type I IFNs production through interaction between its NACHT domain and HSP90, which exerted anti-viral activity in macrophage. These findings shed light on the function and potential regulatory mechanism of NLRP12 in DENV infection, and held the implications for the clinical treatment of DENV and other flavivirus infection disease.

## Data Availability Statement

The original contributions presented in the study are included in the article/**Supplementary Material**. Further inquiries can be directed to the corresponding authors.

## Author Contributions

XL, JP, and YW conducted the experiments. WS, LL, and XH designed research studies. XL, ZD, YL, GJ, and YW analyzed data and wrote the manuscript. All authors contributed to the article and approved the submitted version.

## Funding

This work was supported by grants from National Natural Science Foundation of China (82072062), National Science and Technology Key Projects for Major Infectious Diseases (2017ZX10302301-002), Guangzhou Science and Technology Planning Project (201704020226), The Three Major Scientific Research Projects of Sun Yat-sen University (20200326236), Guangdong Scientific and Technological Research Project for COVID-19 containment (2020A111128022, 2020B111112003), Guangdong Scientific and Technological Research for COVID-19 (202020012612200001), Zhuhai Scientific and Technological Research Project for COVID-19 containment (ZH22036302200029PWC).

## Conflict of Interest

The authors declare that the research was conducted in the absence of any commercial or financial relationships that could be construed as a potential conflict of interest.

## Publisher’s Note

All claims expressed in this article are solely those of the authors and do not necessarily represent those of their affiliated organizations, or those of the publisher, the editors and the reviewers. Any product that may be evaluated in this article, or claim that may be made by its manufacturer, is not guaranteed or endorsed by the publisher.

## Abbreviations

NLRP12, NOD-like receptor 12; DENV, dengue virus; DHF, dengue hemorrhagic fever; DSS, dengue shock syndrome; HSP, heat shock protein; ZIKV, Zika virus; JEV, Japanese Encephalitis virus; YFV, Yellow Fever virus; PRDM1, positive regulatory domain 1; IL-6, interleukin 6; IL-1β, interleukin 1β; TNF-α, tumor necrosis factor-alpha.
