# Supplementary material for: A Novel Role for the Regulatory Nod-Like Receptor NLRP12 in Anti-Dengue Virus Response
Source: Front Immunol. 2021 Dec 9;12:744880. doi: 10.3389/fimmu.2021.744880 (PMC8695442; doi:10.3389/fimmu.2021.744880)
Supplement: Supplementary file 1 [file DataSheet_1.docx]

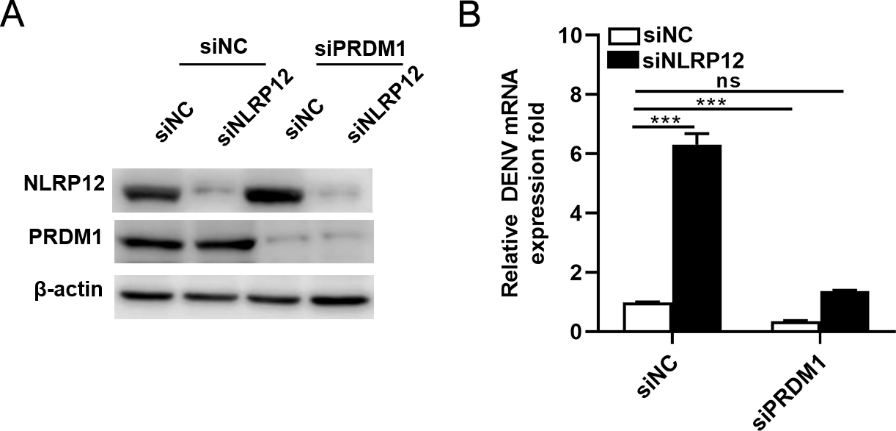
Supplementary Material

**Supplementary figure1. PRDM1 promoted DENV replication via inhibiting NLRP12 expression.** dTHP1 cells were transfected with siNC or siRNAs (10 nM) against PRDM1 or NLRP12 for 24h, then a portion of the cells were collected for western blot to detect the knockdown and overexpressing efficiency. Then the rest of treated cells were infected with DENV with a MOI of 4 for 72h.Then the infected cells were collected for qPCR assay. Each experiment was repeated a minimum of three times and the statistical significance was determined with analysis of one-way analysis of variance (ANOVA). ^***^ P < 0.001.


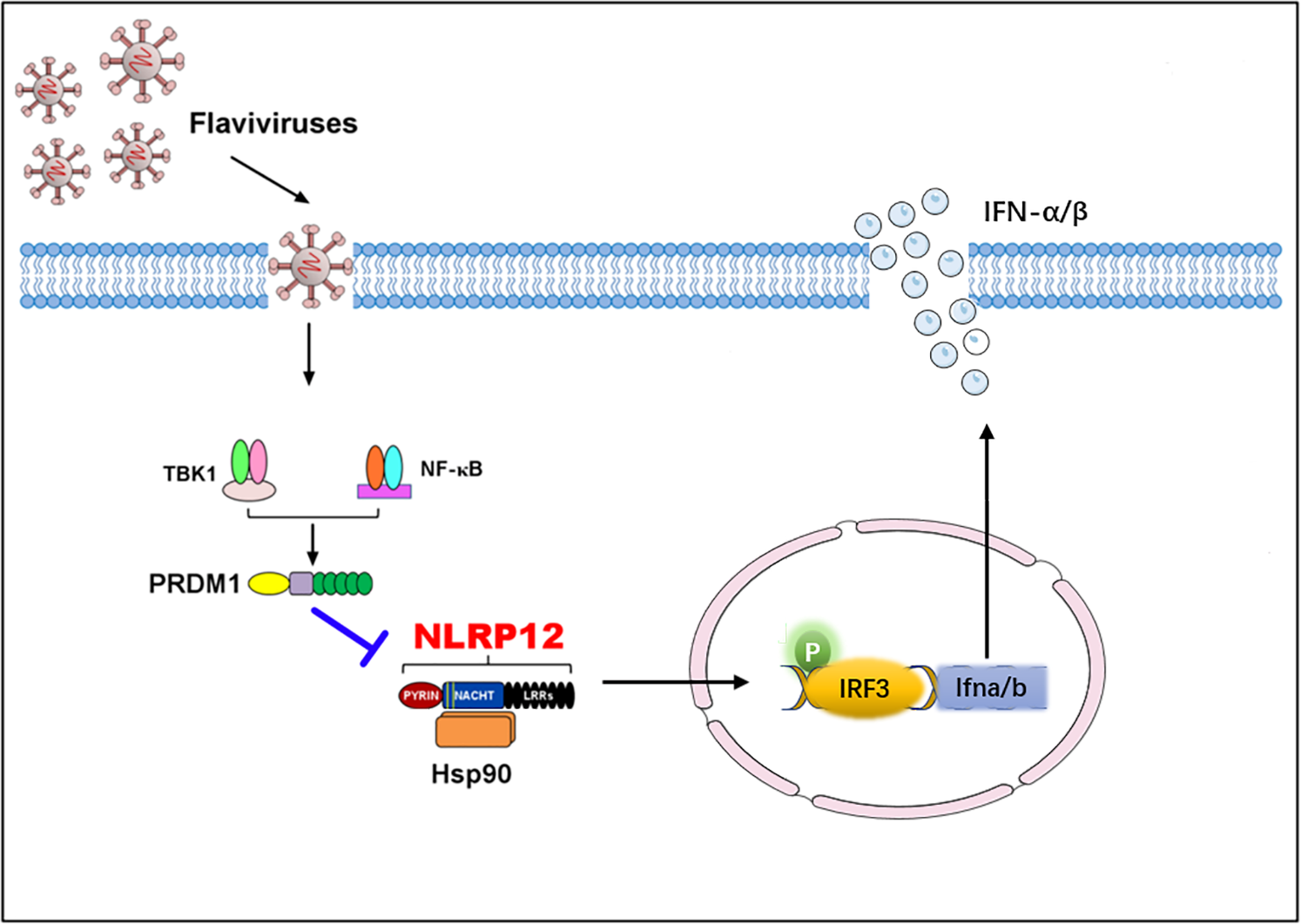


**Supplementary figure2. Scheme of the contribution of NLRP12 to DENV infection.** In DENV infection, PRDM1 expression was increased and suppressed NLRP12 expression, which was dependent on TBK-1/IRF3 and NF-κB signaling pathways. NLRP12 inhibited DENV replication, which relied on the well-conserved nucleotide binding structures of its NACHT domain. Furthermore, NLRP12 interacted with HSP90 dependent on its Walker A and Walker B sites which exerted an antiviral effect. In addition, NLRP12 triggered type I interferon (IFN) production during which HSP90 acted synergistically.
